# Effective Nephroprotection Against Acute Kidney Injury with a Star-Shaped Polyglutamate-Curcuminoid Conjugate

**DOI:** 10.1038/s41598-020-58974-9

**Published:** 2020-02-06

**Authors:** Gina Córdoba-David, Aroa Duro-Castano, Regiane Cardoso Castelo-Branco, Cristian González-Guerrero, Pablo Cannata, Ana B Sanz, María J. Vicent, Alberto Ortiz, Adrián M. Ramos

**Affiliations:** 1 Laboratory of Nephrology, IIS-Fundación Jiménez Díaz, School of Medicine, UAM, Madrid, Spain; 2 Polymer Therapeutics Lab, Centro de Investigación Príncipe Felipe, Valencia, Spain; 3 Pathology, IIS-Fundación Jiménez Díaz, School of Medicine, UAM, Madrid, Spain; 4 Red de Investigación Renal (REDINREN), Madrid, Spain

**Keywords:** Drug discovery, Acute kidney injury

## Abstract

The lack of effective pharmacological treatments for acute kidney injury (AKI) remains a significant public health problem. Given the involvement of apoptosis and regulated necrosis in the initiation and progression of AKI, the inhibition of cell death may contribute to AKI prevention/recovery. Curcuminoids are a family of plant polyphenols that exhibit attractive biological properties that make them potentially suitable for AKI treatment. Now, in cultured tubular cells, we demonstrated that a crosslinked self-assembled star-shaped polyglutamate (PGA) conjugate of bisdemethoxycurcumin (St-PGA-CL-BDMC) inhibits apoptosis and necroptosis induced by Tweak/TNFα/IFNγ alone or concomitant to caspase inhibition. St-PGA-CL-BDMC also reduced NF-κB activation and subsequent gene transcription. *In vivo*, St-PGA-CL-BDMC prevented renal cell loss and preserved renal function in mice with folic acid-induced AKI. Mechanistically, St-PGA-CL-BDMC inhibited AKI-induced apoptosis and expression of ferroptosis markers and also decreased the kidney expression of genes involved in tubular damage and inflammation, while preserving the kidney expression of the protective factor, Klotho. Thus, due to renal accumulation and attractive pharmacological properties, the application of PGA-based therapeutics may improve nephroprotective properties of current AKI treatments.

## Introduction

Acute kidney injury (AKI) involves a sudden and generally transient loss of glomerular filtration leading to the retention of damaging nitrogenous byproducts normally excreted in the urine. Independently of its etiology, AKI usually implies extensive renal parenchymal injury, which, in the case of unsuccessful repair, favors the progression of AKI to chronic kidney disease (CKD). The mortality of AKI may be as high as 50%, and currently, there exist no specific therapies to attenuate AKI or accelerate recovery beyond dialysis^1^. Thus, there is an unmet clinical need for novel AKI treatments.

During the initial stages of AKI, proximal renal tubular cell death triggers a cascade of damaging events, including inflammation, that amplifies kidney injury^2,3^. Thus, the control and execution of apoptosis and several kinds of regulated necrosis depend on environmental conditions as well as on the recruitment of intracellular cell death pathways. Distinct forms of cell death are amenable to pharmacological modulation. Strategies to specifically inhibit caspases, the final executioners of apoptosis, decrease apoptosis, inflammation, and improve renal function in experimental ischemia/reperfusion or septic AKI^4,5^. However, the inhibition of overall caspase activity failed to improve nephrotoxic AKI^6^. In this regard, caspase activation is involved in cellular events beyond cell death regulation, including, among others, cell cycle and the regulation of proliferation^7,8^. Strategies that interfere with initial or intermediate regulators of caspase activity, like protein kinases, survival factors, cytokine death receptors, oxidative stress factors, apoptosome factors, and tumor-suppressor, cell cycle and heat shock proteins, have been shown to decrease the severity of AKI^2^. Additionally, molecules involved in the regulation of necrosis, including necroptosis, pyroptosis, and ferroptosis, are also involved in AKI initiation and progression^6,9,10^.

Curcuminoids are a family of natural compounds derived from the herb *Curcuma longa*; the extracted mixture (turmeric powder) mostly comprises curcumin and copurified demethoxycurcumin and bisdemethoxycurcumin (BDMC). Curcuminoids have multiple potentially beneficial actions, such as preventing parenchymal cell death, inflammation, abnormal cell proliferation, and fibrosis through NF-κB inhibition and antioxidative effects^11^. Indeed, curcumin protects from murine AKI induced by ischemia/reperfusion, glycerol-induced rhabdomyolysis, and cisplatin nephrotoxicity^12,13^. BDMC also prevents kidney fibrosis by activating fibroblast apoptosis^14^. Despite their efficacy and safety profile, the extremely low bioavailability of curcuminoids, resulting from a combination of poor solubility and low stability, limits their therapeutic application^15^. Moreover, maintaining the beneficial pharmacological properties of curcuminoids when introducing chemical modifications in their molecular architecture to improve bioavailability remains a critical challenge.

Nanotechnology-based drug carriers, including polymeric micelles, liposomes, polymeric nanoparticles, nanoemulsions, nanosuspensions, and polymer conjugates can enhance the availability and effective therapeutic outcomes of curcuminoids^16,17^. Specifically, polymer therapeutics are “new chemical entities” (nanomedicines with demonstrated clinical benefits) that can overcome the low bioavailability of free drugs through an increased capacity to i) cross biological barriers, ii) reach specific intracellular compartments and iii) optimize pharmacokinetics^18,19^. Importantly, carboxylated polymers exhibit renal targeting properties and therefore represent a means to target nephroprotectants to the kidney^20,21^. In particular poly-L-glutamic acid (PGA) possesses additional advantages such as excellent water solubility, biocompatibility, non-immunogenicity, biodegradability, and multivalency (allowing dual targeting or drug combination therapy)^22^.

PGA homopolymers, star-shaped PGAs, and crosslinked-star-shaped PGAs purified through covalent capture of self-assembled nanostructures modulate cellular uptake and PK^23,24^. Also, these carriers present a high control on the polymerization process as well as the possibility of post-polymerization with drugs as well as targeting residues^25^, increasing, if needed, the potential of directed molecule design to improve their renal targeting capacity. Herein, we have now assessed the nephroprotective potential of a crosslinked self-assembled star-shaped polyglutamate-BDMC (St-PGA-CL-BDMC) conjugate^23,24^ in experimental cellular and animal models of AKI. We discovered the superior nature of St-PGA-CL-BDMC when compared to the “free” form of BDMC with regards to protection in cultured cells treated with lethal proinflammatory stimuli and experimental murine nephrotoxic AKI models.

## Materials and Methods

### St-PGA-CL-BDMC synthesis

The crosslinlinked star-shaped polyglutamate (St-PGA-CL) polymer was synthesized according to a previously published protocol^23^. The molecular weight of poly (gamma benzyl-L-glutamates) was 31.5 kDa, and the polydispersity index (PDI) 1.26. The polymer was benzyl deprotected using trifluoroacetic acid (TFA)/Hydrogen bromide (HBr) as previously described and then modified with propargylamine (13% mol glutamic acid units GAU) and oligoethylene glycol azide (7% mol GAU units) by post-polymerization modification as described previously^25,26^. The self-assembly and crosslinking of the polymer were performed following recently reported procedures^24^ (See Tables S1–3 for further details on carrier characterization).

Conjugation of BDMC was carried out using the acid form of the St-PGA-CL and proceeded as follows. Briefly, in a two-necked round bottom flask, fitted with a stirrer bar and two septae, the star polymer was dissolved in 10 mL of anhydrous dimethylformamide (DMF) under nitrogen atmosphere. After that, 0.3 eq. of the GAU of DIC (diisopropylcarbodiimide) was added in 5 mL of anhydrous DMF. The reaction was left to proceed for 20 minutes. Then, 0.2 eq. of the GAU of BDMC was added to the reaction mixture, followed by a catalytic amount of DMAP. The pH was maintained around 7. The reaction was then left to proceed for 72 hours. For purification, the mixture was poured into a large excess of diethyl ether. The polymer conjugate was purified using LH-20 column in DMF. After isolation, the yellowish solid was converted into sodium salt form by the careful addition of NaHCO_3_ (1 M). Then, the aqueous solution was washed with diethyl ether till no yellowish coloration was found in the organic phase. Finally, the aqueous phase product was purified by dialysis using Vivaspin® MWCO 5000 and then freeze-dried. BDMC content was determined by UV-VIS at 405 nm using a calibration curve with free BDMC. Yield: 90% BDMC content 8.9% mol GAU, 14.9% wt (See Materials and Methods for further details on St-PGA-CL-BDMC characterization).

### Animal models

All the procedures on animals were performed according to the European Community and Animal Research Ethical Committee guidelines. The animal protocols were approved by the Instituto de Investigación Sanitaria Fundación Jiménez Díaz Animal Research Ethical Committee (body authorized by the Dirección General de Medioambiente, Consejería de medioambiente y Ordenación del Territorio, Comunidad de Madrid, RD 53/2013). AKI was induced in female C57BL/6 wild type mice (10–12 weeks old) employing a single i.p. dose of 250 mg/kg folic acid (FA). Experimental FA-AKI recapitulates the sequence of pathological as well as adaptive responses during human AKI, including several cell death programs involved in apoptosis and regulated necrosis^6,9,27^. Some mice also received a retro-orbital injection of St-PGA-CL-BDMC (4 mg drug equivalent/kg) under anesthesia, 4 h before FA administration. Control mice received the FA vehicle (0.3 M sodium bicarbonate, i.p.). Mice were euthanized under anesthesia (ketamine/xylazine) 48 h after FA administration, corresponding to the acute stage of AKI, characterized by intense cell death accompanied by inflammation and renal dysfunction. Creatinine and urea plasma levels were measured at the institutional Central Laboratory. Kidneys were perfused *in situ* with cold saline before removal. One kidney was snap frozen in liquid nitrogen for RNA and protein studies and the other fixed and paraffin-embedded for immunohistochemistry.

### Cell culture

MCT cells, a cultured line of mouse proximal tubular epithelial cells^28^, were grown in RPMI 1640 (GIBCO, Grand Island, NY) supplemented with 10% decomplemented fetal bovine serum (DFBS), 2 mM glutamine, 100 U/mL penicillin and 10 mg/mL streptomycin, in 5% CO_2_ at 37 °C. For experiments, cells were first arrested using serum-free medium, and subconfluent cells stimulated with a mixture of cytokines containing 100 ng/ml human TWEAK (Merck-Millipore), 30 ng/ml TNFα, 30 U/ml interferon-γ (INFγ, PeproTech). In some experiments, cells were pre-treated with free curcumin (1 mM stock solution in DMSO) (Sigma-Aldrich, Merck), free BDMC (10 mM stock solution in DMSO) (TCI Europe), or St-PGA-CL-BDMC. St-PGA-CL-BDMC dosing was based on the BDMC batch loaded into the conjugate to calculate the drug equivalents in the assay fraction.

### Cell death assays

For assessment of the overall death rate, cells were washed with PBS following stimulation and then incubated with 0.5 mg/ml MTT (Sigma, Merck) for 1 h at 37 °C to detect changes in the metabolic activity. After this step, the MTT solution was withdrawn, and cells allowed to air dry. Finally, deposits of reduced MTT were dissolved with DMSO, and their absorbance read at 570 nm. To assess the degree of plasma membrane damage, cells were seeded in 96 well plates (6000 cells/well). Following incubation, citotoxicity/cytolysis was based on measurements of lactate dehydrogenase (LDH) activity released from the cytosol of damaged cells into the culture supernatant after reduction of tetrazolium salt (pale yellow) to formazan salt (red) and colorimetric detection (Cytotoxicity Detection Kit^PLUS^ (LDH), Roche). For assessment of apoptosis by flow cytometry, remnant adhered cells after treatment were pooled with spontaneously detached cells, centrifuged, and washed with PBS and then incubated in 100 mg/ml propidium iodide, 0.05% Nonidet P-40, and 10 mg/ml RNase A in phosphate-buffered saline (PBS) at 4 °C for more than 1 h. After this step, cells were centrifuged, and the cellular pellet suspended in PBS. The number of apoptotic cells with decreased DNA staining (G_0_ hypodiploid cells) was counted by flow cytometry using BD CellQuest Software (BD Biosciences) and the percentage of apoptosis calculated in relation to the total number of cells. *In vivo* cell death was assessed by TUNEL assay performed in 3 µm-thick sections of paraffin-embedded renal tissue (ApopTag®Peroxidase *in Situ* apoptosis detection kit, Millipore, Merck) according to the manufacturer’s instructions.

### Western blotting

Total protein extracts were prepared by homogenizing samples in lysis buffer (50 mmol/L Tris, 150 mmol/L NaCl, 2 mmol/L EDTA, 2 mmol/L EGTA, 0.2% Triton X-100, 0.3% NP-40, 0.1 mmol/L PMSF, 25 mmol/L NaF). For nuclear extracts, the NE-PER^TM^ nuclear and cytoplasmic extraction kit (Pierce, Thermofisher Scientific) was used according to the manufacturer’s instructions. Proteins were separated by SDS-PAGE under reducing conditions and then blotted onto nitrocellulose membranes. Membranes were blocked with 5% defatted milk in TBS-T (0.05 mol/L Tris, 0.15 mol/L NaCl, 0.05% Tween 20, pH 7.8). Thereafter, membranes were probed overnight at 4 °C with primary antibodies in the same blocking solution or 5% BSA in TBS-T and then incubated with secondary HRP-conjugated antibodies for 1 h at room temperature. Primary antibodies were: phospho-MLKL (1/500; ab196436; Abcam, Inc.); cleaved caspase-8 (1/1000, 8592, Cell Signaling Technology); p-cJUN (1/1000, 3270, Cell Signaling); cleaved IL-33 (1/1000; AF3626; R&D Systems), heme oxygenase-1 (HO-1, 1/2000, ADI-OSA-150-D, Enzo) and p65 (1/1000, 8242, Cell Signaling Technology). Total protein content for loading controls was assessed with Ponceau Red or by means of the fluorescence incorporated into tryptophan amino acids of proteins samples ran in Stain-Free^TM^ gels (BioRad).

### Immunofluorescence

Cells were fixed in 4% paraformaldehyde/PBS, permeabilized in 0.1% Triton X-100/PBS, washed in 1% BSA/PBS, blocked with 4% BSA/PBS, and stained with rabbit polyclonal anti-p65 (1:200, sc-8008, Santa Cruz Biotechnology). Cells were incubated with Alexa secondary antibodies (Invitrogen) and nuclei counterstained with propidium iodide or 4′,6-diamidino-2-phenylindole (DAPI). Cells were analyzed using a Confocal System TCS SP5 (Leica).

Cell uptake of St-PGA-CL-BDMC was assessed by live fluorescence imaging in microscopy chambers (1 µ-slides) of cultured cells treated with 10 µM St-PGA-CL-BDMC for 6 h. After stimulation, cells were washed with PBS-BSA 0.1% and then incubated for 20 min with the lysosomal marker LysoTrackerTMGreen DND-26 (Invitrogen). After washing with PBS-BSA 0.1%, cells were left in culture medium and placed in the microscope culture chamber (37 °C, 5% CO_2_) and analyzed using the same confocal system.

### Gene expression

Total RNA was extracted using Tripure (Roche), and 1 μg was reverse transcribed with the High-Capacity cDNA Archive Kit (Applied Biosystems, Thermofisher Scientific). Quantitative PCR was performed in a 7500 Real-Time PCR System with Prism 7000 System SDS Software (Applied Biosystems). RNA expression was corrected for endogenous glyceraldehyde-3-phosphate dehydrogenase (GAPDH) mRNA levels. Fluorogenic (FAM or VIC) predesigned primers were from Applied Biosystems.

### Biochemical and histological studies

Creatinine and Urea plasma levels were assessed by biochemical methods intended for automatic measurements in a biochemistry analyzer (Roche/Hitachi cobas® c701/702) based on the enzymatic decomposition with creatininasa and ureasa, respectively, then followed by colorimetric detection of the reactions products.

Tubular injury was assessed in kidney tissue sections stained with hematoxylin–eosin by a renal pathologist (P.C) blinded as to the nature of the samples. Evidence of tubulointerstitial injury including pyknosis/apoptosis, activity, proliferation/regeneration, flattening, necrosis, calcifications and intratubular precipitations were individually scored on a semiquantitative scale from 0 to 3. Results from each item were added to yield the overall tubular injury score (maximal value 21) as previously described^29^.

For immunohistochemistry, endogenous peroxidase was blocked and then sections were incubated overnight at 4 °C with the following primary antibodies: PCNA (1:50; sc-7907, Santa Cruz); active caspase-3 (1/100, G748A, Promega); phospho-cJUN (1/250, 3270, Cell Signaling) or 4-hydroxynonenal (4-HNE) (1:400; ab46545; Abcam). Finally, sections were washed, stained with 3,3′-diaminobenzidine (DAB) as chromogen (Dako, Denmark), counterstained with Carazzi’s hematoxylin, dehydrated and mounted in DPX medium (Merck, NJ, USA).

### Statistical analysis

Statistical analysis was performed using SPSS 11.0 (SPSS), expressing the results as mean ± SD. Significance (*p* < 0.05) was assessed by the non-parametric Mann–Whitney test for two independent samples.

## Results

### Rational design of renal targeted curcuminoid conjugates and interaction with tubular cells

**Figure 1:**
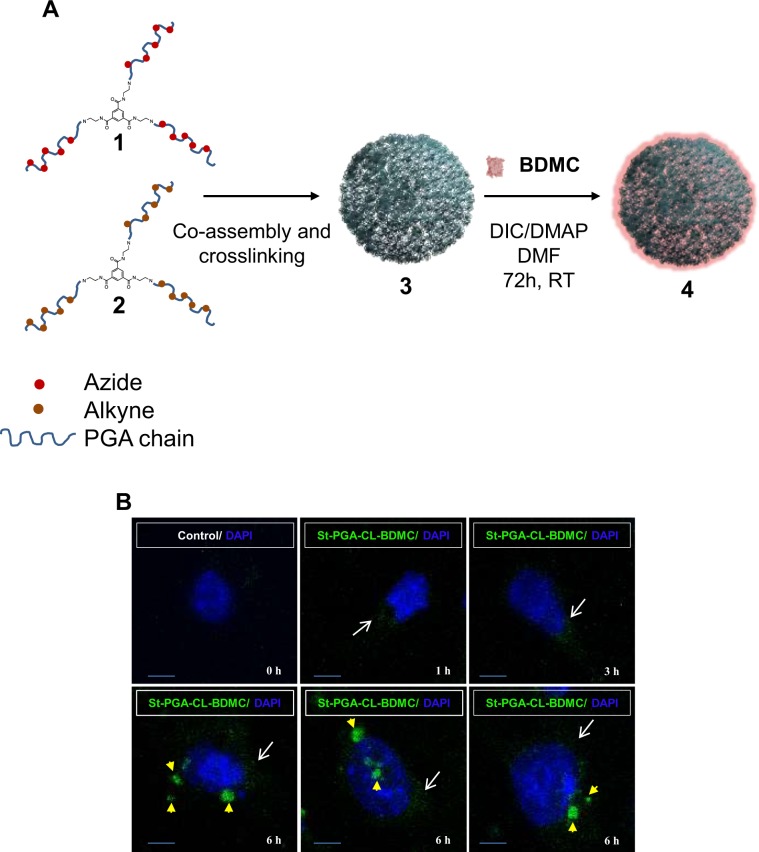
St-PGA-CL-BDMC synthesis and main physicochemical characteristics. (**A**) Schematic representation of the synthesis of St-PGA-CL-BDMC (4) by means of bisdemethoxycurcumin (BDMC) conjugation to St-PGA-CL (3) after azide (1) and alkyne (2) bearing St-PGAs co-assembly and covalent capture by copper catalyzed alkyne-azide cycloaddition. (**B**) Confocal immunofluoresce in murine tubular cells (MCT) treated with 10 µM St-PGA-CL-BDMC (green fluorescence) for 1 to 6 h to analyze the intracellular uptake and transit of the conjugate. Nuclei were counterstained with DAPI (blue fluorescence). White arrows indicate the captured conjugate at early time points (1–3 h) and yellow arrowheads point to the conjugate inside lysosomes at a later time points (6 h). Control cells that were left untreated displayed a faint autofluorescence. Original magnification x200. Scale bar 25 µM.

We synthesized a novel family of biodegradable St-PGA-CL-BDMC conjugates to enhance solubility and promote renal targeting of BDMC while also improving BDMC pharmacokinetics. We conjugated BDMC to St-PGA-CL polymeric carrier employing post-polymerization modification approaches^25^, due to the reported renal accumulation after intravenous administration^23,24^. We carried out the conjugation of BDMC by the carbodiimide-mediated activation of St-PGA-CL catalyzed by DMAP. After purification, we detected a St-PGA-CL-BDMC conjugate (90% yield) with a total BDMC loading of 8.9 mol% (14.9 wt%), a size around 40 nm, and a clear negative Z potential (Fig. 1, Table 1, and Tables S2 and S3).

**Table 1:** St-PGA-CL-BDMC characterization including drug loading, size and zeta potential.

Compound	wt% BDMC^a^	Size (R_h_, nm)^b^	Z-pot (mV)^c^
**St-PGA-CL-BDMC**	14.9%	40.2 ± 2.4	−38.6 ± 2.2

^a^Determined by UV-VIS at 405 nm.

^b^Determined by dynamic light scattering techniques using a conjugate solution of 1 mg/mL in ddH_2_O. Size expressed in number mean.

^c^Determined using Zetasizer using a conjugate solution of 1 mg/mL in 1 mM solution of KCl.

To study the uptake of St-PGA-LC-BDMC by cultured tubular cells, we took advantage from the intrinsic fluorescence of BDMC to monitor its uptake and intracellular delivery over time. St-PGA-LC-BDMC was taken up by tubular cells in a time-dependent fashion from 1 h onward and with a maximum visualization at 6 h thus demonstrating that the conjugate may permeate the cytoplasmic membrane. In addition, the fluorescence pattern changed from punctuated and distributed all over the cytoplasm to aggregates with mainly perinuclear location, suggesting lysosomal targeting **(**Fig. 1B**)**. Indeed, lysosomal delivery of St-PGA-LC-BDMC at 6 h was confirmed in living cells by colocalization with acidic compartments stained with the fluorescent dye Lysotracker Green **(**Supplentary Fig. S1**)**.

### St-PGA-CL-BDMC displays potent antiapoptotic effects in cultured renal tubular cells

**Figure 2:**
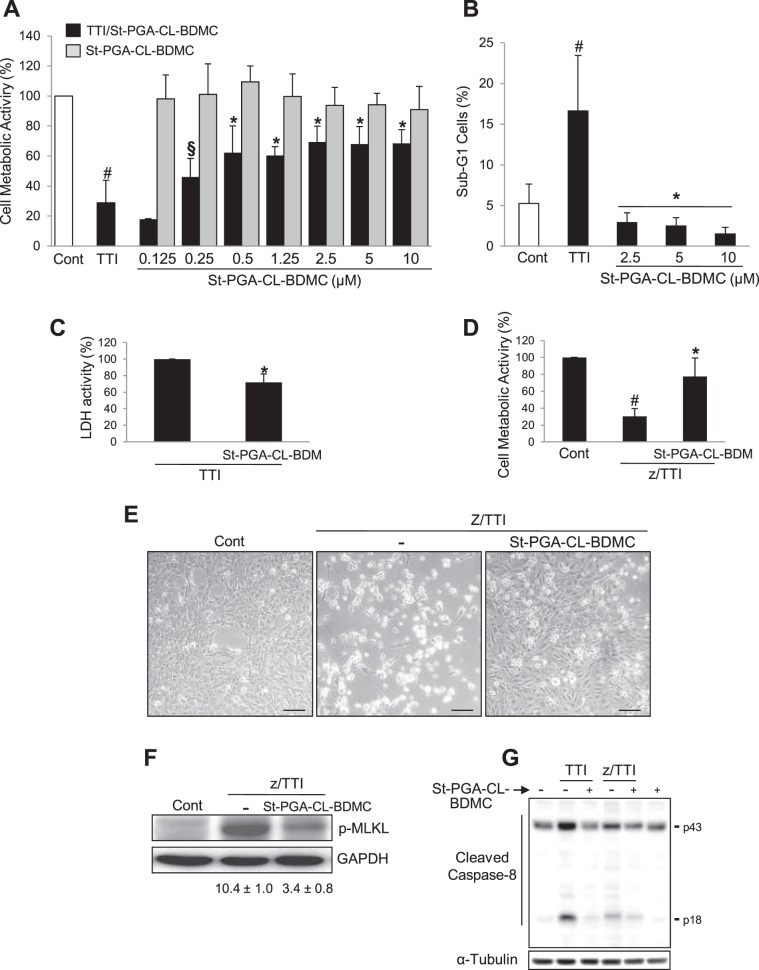
St-PGA-CL-BDMC inhibits apoptosis and necroptosis in cultured renal tubular cells. (**A**) Percentage of cell viability according to the metabolic activity assessed by the MTT assay in cultured murine MCT tubular cells stimulated for 24 h with 100 ng/ml Tweak/30 ng/ml TNFα/30 U/ml INFγ (TTI) alone or following pretreatment with variable doses of 10 µM St-PGA-CL-BDMC for 1 h before stimulation. ^#^p < 0.001 vs control; ^§^p < 0.05 and *p < 0.001 vs TTI (n = 5–10 independent experiments). (**B**) Percentage of haplodiploid apoptotic cells corresponding to the cell population distributed along the sub-G1 region of the cell cycle identified by flow cytometry of cell DNA content. Cells were subjected to TTI alone under the same conditions as in A or pretreated with the indicated doses of 10 µM St-PGA-CL-BDMC. (**C**) Percentage of LDH activity assessed in cell culture supernatants treated as in A. In every individual experiment, the activity of supernanant LDH in cells incubated with St-PGA-CL-BDMC and TTI was expressed as a percentage of the activity in TTI-exposed cells considered to be 100%. *p < 0.01 vs TTI (n = 5). (**D**) Percentage of cell viability inferred from the metabolic activity assessed by the MTT assay in tubular cells stimulated for 8 h with 20 µM zVAD-fmk plus TTI (z/TTI) alone or following pretreatment with 10 µM St-PGA-CL-BDMC for 1 h before stimulation. ^#^p < 0.05 vs control; *p < 0.05 vs TTI (n = 4). (**E**) Representative contrast phase microphotographs of tubular cells left untreated (Control) or stimulated with z/TTI alone or in the presence of 10 µM St-PGA-CL-BDMC (original magnification 200x, scale bar 200 µm). The figure shows that St-PGA-CL-BDMC markedly decreased z/TTI-induced morphological changes and detachment. Results are presented as mean ± SD. (**F**) Phosphorylated MLKL protein levels (p-MLKL) in cultured MCT cells following 20 µM z-VAD-fmk plus TTI (z/TTI) or 10 µM St-PGA-CL-BDMC/zTTI stimulation. The figure shows a representative Western blot and the corresponding band quantification (n = 2 independent experiments). (**G**) Representative western blot of cleaved caspase-8 in MCT cells exposed to TTI or z/TTI alone or following pretreatment with 10 µM St-PGA-CL-BDMC (n = 2 experiments with similar results).

We stimulated murine MCT renal tubular cells with a combination of proinflammatory cytokines (Tweak/TNFα/INFγ, hereafter named as TTI) previously shown to induce apoptosis^30^, in the presence of St-PGA-CL-BDMC or vehicle to explore a potential role for our polymer-drug conjugate in the prevention of renal cell death. The MTT assay established that TTI treatment induced a ~70% loss in cell metabolic activity/viability compared to untreated control cells at 24 h **(**Fig. 2A**)**. Encouragingly, we discovered a significant attenuation of cell metabolic activity/viability loss following treatment with St-PGA-CL-BDMC (54% decrease at 0.25 µM eq., reaching a plateau of 31% decrease at 0.5 µM St-PGA-CL-BDMC eq.) **(**Fig. 2A**)**.

In a second set of experiments using flow cytometry analysis, we discovered a significantly lower percentage of apoptotic hypodiploid cells induced by TTI following cotreatment with 2.5–10.0 µM St-PGA-CL-BDMC eq., thus confirming antiapoptotic properties of St-PGA-CL-BDMC **(**Fig. 2B**)**. Moreover, LDH activity in culture supernatants of tubular cells stimulated with TTI was partially but significantly inhibited by St-PGA-CL-BDMC, meaning that the BDMC conjugate decreased TTI-induced membrane permeabilization and therefore necrosis **(**Fig. 2C**)**. Furthermore, we explored whether St-PGA-CL-BDMC may also inhibit necroptosis, a main type of regulated necrosis actively involved in AKI. We have previously shown that cotreatment of renal tubular cells with the general caspase inhibitor z-VAD-fmk in combination with TTI (z/TTI) switches the mechanism of cell death from apoptosis to necroptosis^30,31^. In our experiments, z/TTI stimulation lowered the metabolic activity/viability of renal tubular cells by ~70% when compared to untreated control cells **(**Fig. 2D**)**. However, results obtained with 10.0 µM St-PGA-CL-BDMC indicated an efficient blockade of the necroptosis pathway by preventing both, the z/TTI-induced loss of metabolic activity/viability and associated morphological changes **(**Fig. 2D,E**)** and the phosphorylation of the protein kinase MLKL, which is considered the final event triggering the necroptosis execution **(**Fig. 2F**)**. We also explored the impact of St-PGA-CL-BDMC on the activation of caspase-8, a key caspase whose state of activation determines the occurrence of apoptosis (when active) or necrosis (when inhibited) **(**Fig. 2G**)**. In tubular cells, TTI promoted caspase-8 cleavage, resulting in the appearance of early (45 kDa) and late (18 kDa) proteolytic fragments. Exposure to z/TTI, which induces necroptosis, inhibited the late activation step of caspase-8 almost completely but did not efficiently prevent the initial processing step resulting in the generation of the 45 kDa fragment. Similar to the necroptosis inhibitor necrostatin, which also inhibits TTI-induced cell death^9^, PG-BDMC prevented caspase-8 processing to 45 and 18 kDa fragments in presence of TTI or z/TTI, thus suggesting that it prevents cell death upstream of caspase-8 activation but contrary to zVAD, it does not trigger necroptosis **(**Fig. 2G**)**.

**Figure 3:**
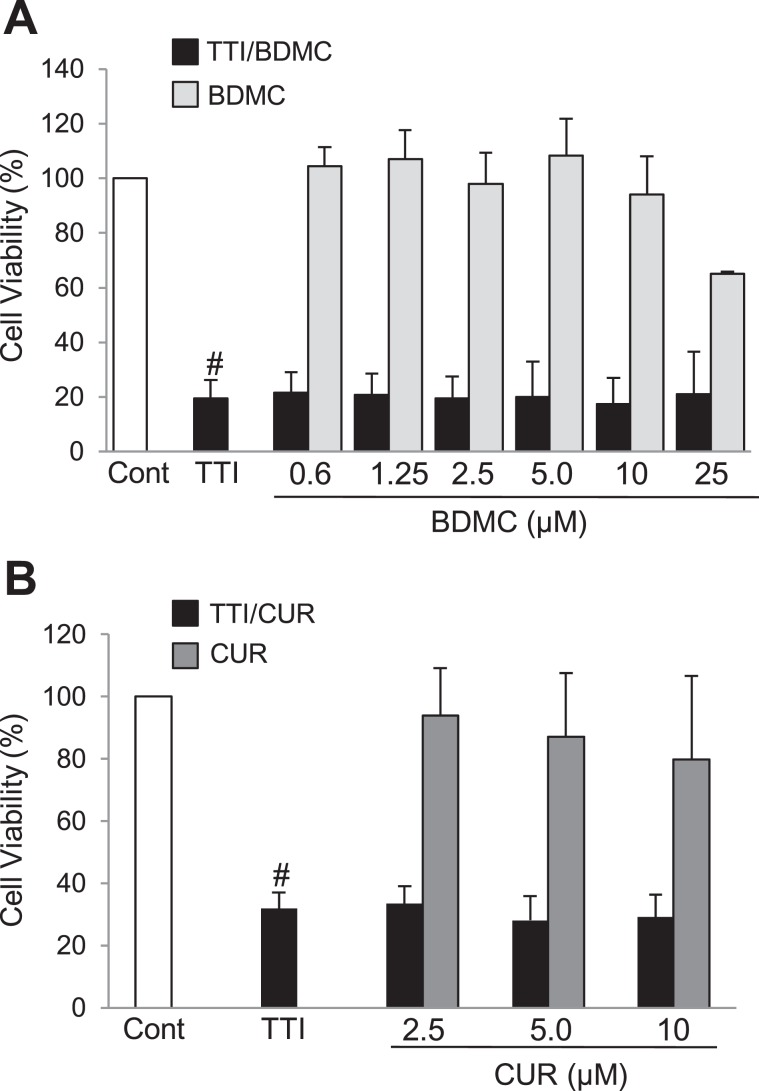
Free BDMC and curcumin do not protect from TTI-induced cell death. Cultured MCT cells were exposed to TTI alone for 24 h or following pre-treatment with free BDMC (**A**) or curcumin (**B**) for 1 h before TTI addition. (**A–B**) The percentage of cell viability was assessed by the MTT assay. Curcuminoids were not toxic by themselves but did not prevent TTI-associated toxicity. Bar charts represent the mean ± SD of 3–5 individual experiments. ^#^p < 0.001 vs control.

Finally, we explored the protective effect of free BDMC and curcumin, chosen as the most representative and widely studied curcuminoid, in tubular cells stimulated with TTI. Neither free BDMC **(**Fig. 3A**)** nor curcumin **(**Fig. 3B**)** displayed toxicity within the same concentration range in which St-PGA-CL-BDMC protected against TTI-induced apoptosis (0.6–10 µM). However, in contrast with St-PGA-CL-BDMC, neither 0.6–10 µM free BDMC nor 2.5–10 µM curcumin offered any protection against TTI-induced cell death **(**Fig. 3A,B**)**. These results demonstrate that conjugation enhances the antiapoptotic potency of the curcuminoid, likely through increased BDMC intracellular bioavailability.

### St-PGA-CL-BDMC inhibits NF-κB activation and downstream gene expression of proinflammatory, tubular stress, and regenerative markers in renal tubular cells

**Figure 4:**
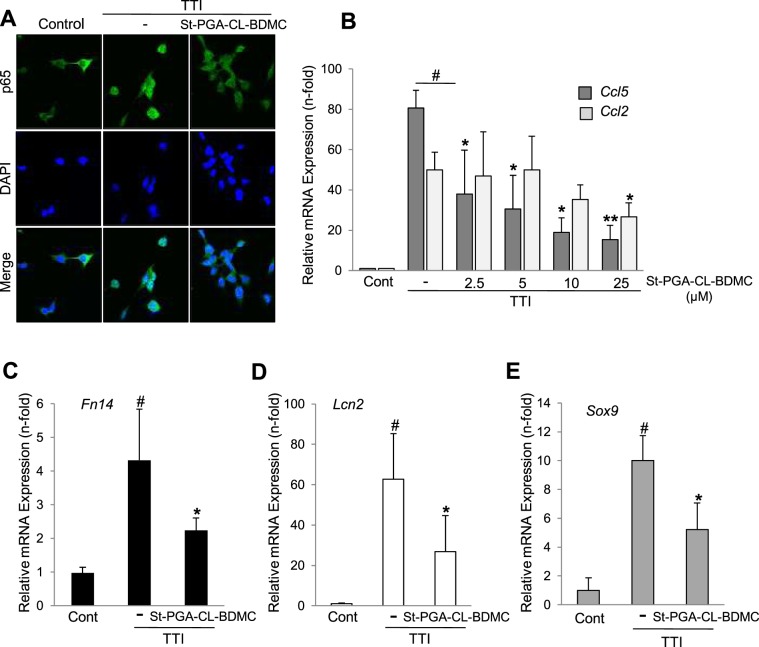
St-PGA-CL-BDMC inhibits TTI-induced NF-κB activation and proinflammatory activity in cultured renal tubular cells. (**A**) MCT cells were stimulated with TTI alone for 30 min or following pretreatment for 1 h with 10 µM St-PGA-CL-BDMC. NF-κB nuclear translocation/activation was assessed by fluorescence immunocytochemistry and confocal microscopy of the RelA/p65 subunit. The figure is a representative experiment showing that TTI induces the nuclear translocation of p65 (green fluorescence) and this is inhibited by St-PGA-CL-BDMC. Nuclei were counterstained with DAPI (blue fluorescence). The experiment was replicated for at least three times. Original magnification x 200. (**B–E**) Relative mRNA expression of NF-κB-dependent genes CCL5 and CCL2 (^#^p < 0.001 vs control; *p < 0.05 vs TTI; **p < 0.01 vs TTI) (**B**), Fn14 (^#^p < 0.001 vs control; *p < 0.05 vs TTI) (**C**); LCNA (^#^p < 0.001 vs control; *p < 0.05 vs TTI) (**D**) and SOX9 (^#^p < 0.001 vs control; *p < 0.05 vs TTI) (**E**) assessed by q-RT-PCR in MCT cells stimulated for 6 h with TTI alone or following pre-treatment with 2.5–25 µM (**B**) or 10 µM St-PGA-CL-BDMC (**C–E**). Results are presented as mean ± SD of at list 3 experiments.

NF-κB activation is a hallmark of the tubular cell stress response during AKI, and its prevention decreases the uncontrolled signaling that amplifies inflammatory events, which in turn promotes cell death^32,33^. Treatment of tubular cells with 10 µM eq. St-PGA-CL-BDMC for 30 min prevented TTI-induced NF-κB/p65 nuclear translocation **(**Fig. 4A**)** and decreased the expression of NF-κB-dependent genes such as those encoding the chemokines Ccl2 (MCP1) and Ccl5 (Rantes) **(**Fig. 4B**)**, the proinflammatory Tweak receptor Fn14 **(**Fig. 4C**)**, the tubular injury marker Lcn2 **(**Fig. 4D**)**, and the renal repair-associated transcription factor Sox9 **(**Fig. 4E**)**.

### St-PGA-CL-BDMC preserves renal function in murine nephrotoxic AKI

**Figure 5:**
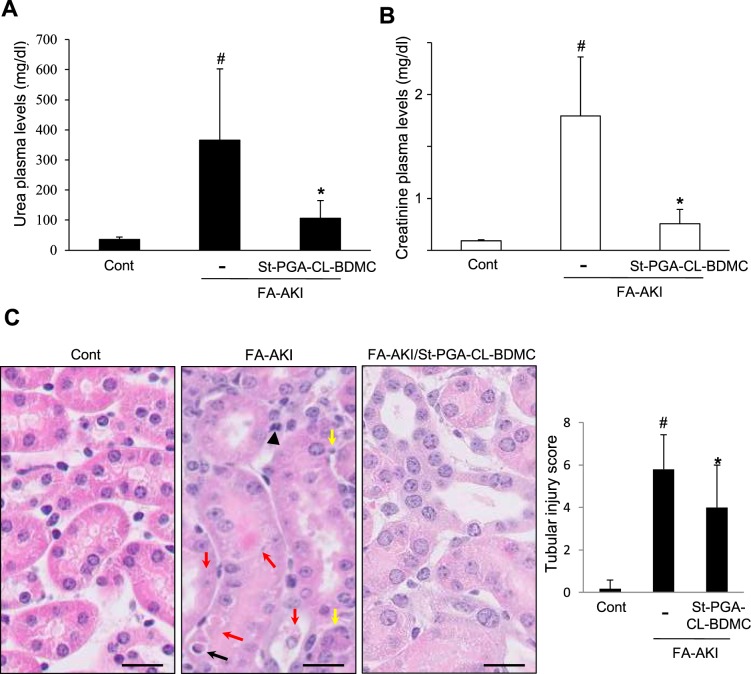
*In vivo* treatment with St-PGA-CL-BDMC prevents renal dysfunction and injury provoked by FA-induced AKI. C57/BL6 mice were injected with vehicle (Cont, n = 7), folic acid (FA-AKI, n = 10), or St-PGA-CL-BDMC and folic acid (FA-AKI + St-PGA-CL-BDMC, n = 10). St-PGA-CL-BDMC significantly decreased the severity of renal dysfunction by lowering the plasma levels of urea **(A)** and creatinine **(B)** induced by AKI. Bar graphs represent the mean ± SD of the entire set of animals in each group for both urea (^#^p < 0.01 vs control; *p < 0.01 vs FA-AKI) and creatinine (^#^p < 0.001 vs control; ^*^p < 0.001 vs FA-AKI). (**C**) St-PGA-CL-BDMC prevents histological evidence of AKI. Kidney tissue sections were stained with H&E (left panel) and the tissue integrity assessed by an injury score (right panel). Representative microphotographs showing typical injury signs in AKI that were decreased by St-PGA-CL-BDMC: apoptotic cells (black arrow), tubular necrosis (cell detachment, intratubular cell debris) (red arrows), mitotic cells (black arrowhead), nuclei size and chromatin compactation heterogeneity (yellow arrows). ^#^p < 0.01 vs control; *p < 0.05 vs FA-AKI. Original magnification 400x. Scale bar 25 µm.

As St-PGA-CL-BDMC prevented critical processes involved in AKI pathogenesis in cultured tubular cells, we next explored the nephroprotective potential of our novel conjugate *in vivo* in mice with FA-AKI. We administered St-PGA-CL-BDMC 4 h before FA dosage. While FA-AKI led to increased plasma urea **(**Fig. 5A**)** and creatinine levels **(**Fig. 5B**)**, however, St-PGA-CL-BDMC treatment significantly prevented against the AKI-induced urea and creatinine raise thus protecting from loss in renal function **(**Fig. 5A,B**)**. AKI was associated to an spectrum of histological tubulointerestitial lesions including extensive areas of necrotic and apoptotic cell death and of tubular cell proliferation that, in accordance with the better renal function, were milder in mice cotreated with St-PGA-CL-BDMC **(**Fig. 5C**) (**Supplementary Fig. S2**)**.

### St-PGA-CL-BDMC prevents tubular injury and maladaptive signaling in murine nephrotoxic AKI

**Figure 6:**
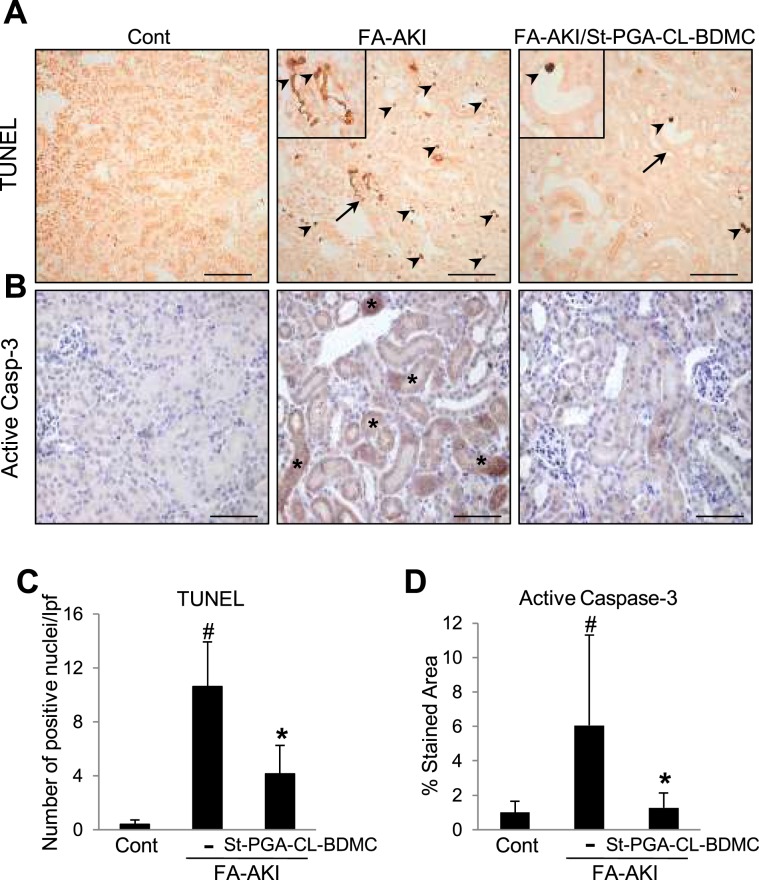
*In vivo* treatment with ST-PGA-CL-BDMC reduces cell death and caspase-3 activation in FA-AKI. Renal cell death was assessed by TUNEL staining (**A,C**) or by active caspase-3 immunohistochemistry (**B,D**) in vehicle-injected mice (Control, n = 7), in mice with FA-AKI (FA-AKI, n = 10) or in mice with FA-AKI cotreated with St-PGA-CL-BDMC (FA-AKI + ST-PGA-CL-BDMC, n = 10). (**A**) St-PGA-CL-BDMC reduces the number of TUNEL positive cells in FA-AKI kidneys. **A** shows representative images corresponding to renal sections from Control, FA-AKI, and FA-AKI + St-PGA-CL-BDMC groups. TUNEL positive nuclei are indicated by arrowheads, while the arrows point areas showed at a higher magnification as detail (inserted images). Original magnification x 200. Scale bar 100 μm. (**C**) shows TUNEL quantification by counting the number of positive nuclei per low power field (lpf). ^#^p < 0.001 vs control; *p < 0.05 vs FA-AKI. (**B,D**) St-PGA-CL-BDMC reduces caspase-3 activation in FA-induced AKI kidneys. **B** shows representative images. Asterisks identify representative tubules with positive staining for the processed active form of caspase-3. Original magnification x200. Scale bar 100 μm. The bar chart in **D** depicts the ratio of the active caspase-3 stained area to total area expressed as a percentage. ^#^p < 0.05 vs control; ^*^p < 0.01 vs FA-AKI. Results are presented as mean ± SD.

Proximal tubular cell death during AKI is a critical event that results in the amplification of kidney injury^3^. *In vivo* evaluation of cell injury and death revealed that tubular injury, indicated by an increased number of TUNEL-stained **(**Fig. 6A**)** and active caspase-3 positive **(**Fig. 6B**)** tubular cells, compared to control animals, characterized FA-AKI. St-PGA-CL-BDMC treatment prevented the increase in TUNEL-positive **(**Fig. 6A,C**)** and caspase-3-positive **(**Fig. 6B,D**)** tubular cells, thus confirming that St-PGA-CL-BDMC inhibits renal tubular cell apoptosis *in vivo*.

**Figure 7:**
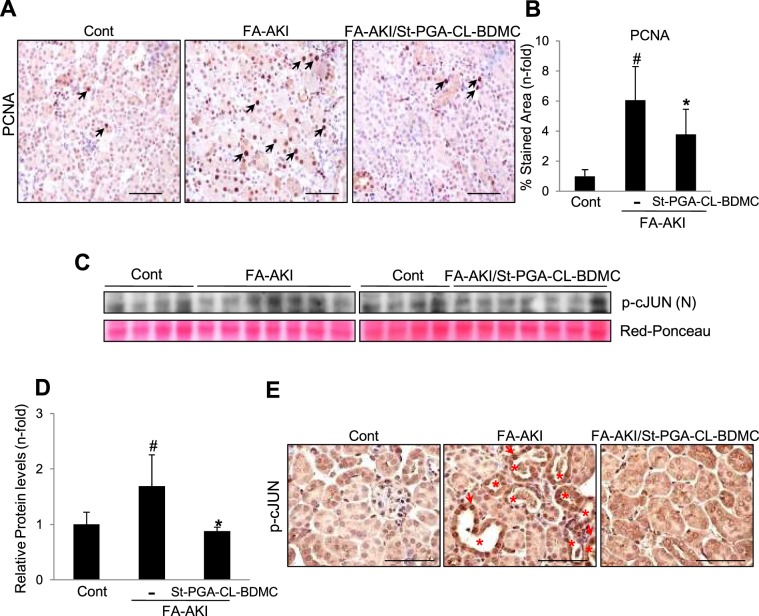
St-PGA-CL-BDMC attenuates reparative and AKI-to-CKD transition mechanisms in FA-AKI. Evaluation of the impact of St-PGA-CL-BDMC treatment on key events governing regeneration or AKI-to-CKD transition in C57/BL6 mice with FA-AKI. Mice were distributed into the following groups: vehicle-treated (control, n = 4–7), FA-AKI (FA-AKI, n = 7–10) and FA-AKI treated with St-PGA-CL-BDMC (FA-AKI + St-PGA-CL-BDMC, n = 7–10). (**A,B**) The proliferation rate estimated by PCNA staining was lower in kidneys from AKI mice treated with St-PGA-CL-BDMC. Representative images of PCNA immunohistochemistry (**A**) and the corresponding quantification in the complete set of animals (**B**). Arrows in **A** show PCNA positive nuclear staining. Original magnification x200. Scale bar 100 μm. ^#^p < 0.001 vs control; *p < 0.05 vs FA-AKI. (**C**) Maladaptive signaling was evaluated by the nuclear levels of phosphorylated cJUN (p-cJUN). Representative western blot the corresponding quantification (**D**) show that compared to control kidneys, the nuclear content of p-cJUN increased in kidneys from FA-AKI mice and St-PGA-CL-BDMC significantly prevented this effect. Specific bands were matched against Ponceau red stained bands used as loading control. ^#^p < 0.001 vs control; *p < 0.05 vs FA-AKI. (**E**) Renal p-cJUN was localized by immunohistochemistry. Images from representative animals for each experimental group showing higher nuclear p-cJUN content in FA-AKI than in control mice or St-PGA-CL-BDMC-treated AKI mice. Asterisks indicate tubules with high p-cJUN expression and arrows point individual positive nuclei. Original magnification x 400. Scale bar 100 μm Results are presented as mean ± SD.

Tubular cell death triggers adaptive mechanisms that eventually lead to recovery of renal structure and function, including tubular proliferation. Accordingly, we observed that St-CL-PGA-BDMC, in addition to reduce tubular apoptosis **(**Fig. 1A–B**)**, also significantly decreased the resulting increase in tubular cell proliferation, as assessed by a lower number of PCNA positive tubular cells **(**Fig. 7A,B**)**. Nuclear phosphorylation/activation of c-JUN is a known driver of maladaptive signaling leading to kidney fibrosis^34^. Compared to untreated FA-AKI mice, kidneys from animals treated with St-PGA-CL-BDMC exhibited lower levels of nuclear phospho-c-JUN as assessed by Western blot **(**Fig. 7C,D**) (**Supplementary Fig. S3**)** and further localized in tubular cells by immunohistochemistry **(**Fig. 7E**)**.

Overall, the reduction in tubular cell injury and death and preserved renal function in association with lower tubular proliferation and the recruitment of signaling pathways involved in both repair and maladaptive responses suggests that St-PGA-CL-BDMC treatment prevents both AKI and the subsequent tissue responses that eventually lead to chronic injury.

We next focused on the impact of St-PGA-CL-BDMC on mediators of tubular cell death and inflammation.

### St-PGA-CL-BDMC inhibits ferroptosis and reduces related oxidative stress markers in FA-AKI

**Figure 8:**
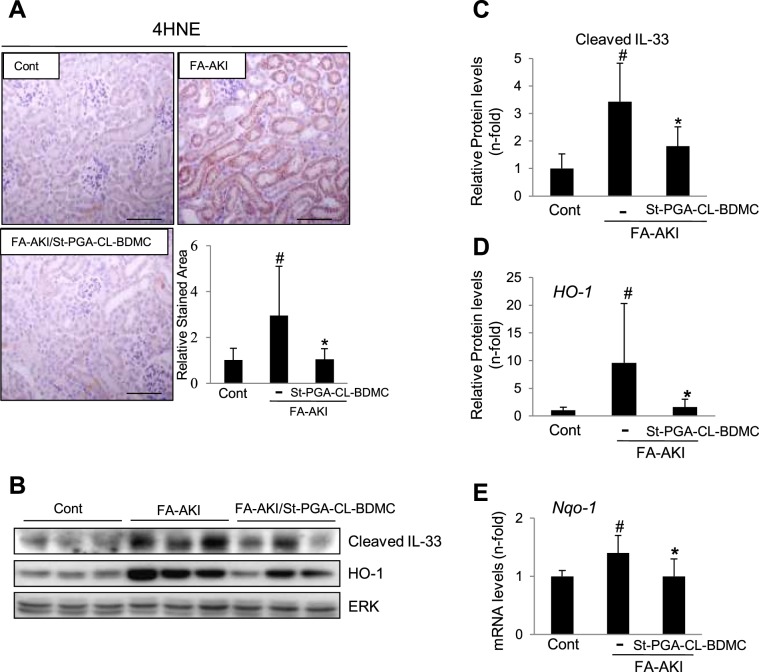
St-PGA-CL-BDMC treatment prevents key renal cell death and oxidative stress pathways in FA-AKI. Groups of animals were the same as in preceding figures. (**A**) Representative IHC images of 4-Hydroxynonenal (4-HNE) staining in mice with AKI and the corresponding quantification including all mice in each experimental group. St-PGA-CL-BDMC decreased FA-AKI-induced 4-HNE staining. ^#^p < 0.001 vs control; *p < 0.05 vs FA-AKI. Original magnification x200. Scale bar 100 μm. (**B**) Representative western blot detection of cleaved IL-33 and heme oxygenase-1 (HO-1) in total kidney protein extracts from control, AKI or AKI/St-PGA-CL-BDMC mice. St-PGA-CL-BDMC prevented kidney IL-33 processing/activation and upregulation of HO-1 expression. (**C,D**) Quantification of IL-33, ^#^p < 0.001 vs control; *p < 0.05 vs FA-AKI **(C)** and HO-1, ^#^p < 0.05 vs control; *p < 0.05 vs FA-AKI. (**D**) western blots including the complete set of animals. (**E**) Nqo-1 mRNA expression assessed by q-RT-PCR in kidneys from control mice and in mice with FA-AKI left untreated or treated with ST-PGA-CL-BDMC. ^#^p < 0.001 vs control; *p < 0.05 vs FA-AKI. Results are presented as mean ± SD.

We recently demonstrated that ferroptosis, a regulated necrosis program, is an essential driver of the initial wave (24–48 h) of tubular cell death in FA-AKI^6^. In association with the above cell death increase at 48 h **(**Fig. 6**)**, analysis of kidneys from FA-AKI mice revealed the development of critical features of ferroptosis, such as increased lipid peroxidation as assessed by increased 4-hydroxynonenal (4-HNE) staining by immunohistochemistry **(**Fig. 8A**)** and IL-33 proteolytic processing to its proinflammatory form as evaluated by Western blot **(**Fig. 8B**) (**Supplementary Fig. S4**)**. Interestingly, St-PGA-CL-BDMC also diminished 4-HNE formation **(**Fig. 8A**)** and the proinflammatory fragmentation of IL-33 **(**Fig. 8B,C**) (**Supplementary Fig. S4**)**.

Lipid peroxidation leading to cell membrane disruption and ferroptosis is a consequence of oxidative stress and reactive oxygen species (ROS) formation. In this regard, FA-AKI kidneys displayed increased mRNA and protein levels of the oxidative stress markers heme oxigenase 1 (HO-1) **(**Fig. 8B,D**) (**Supplementary Fig. S4**)** and NAD(P)H dehydrogenase (quinine 1) (NQO-1) **(**Fig. 8E**)**, which was prevented by St-PGA-CL-BDMC treatment **(**Fig. 8B,D,E**)** in agreement with the observation of lower 4-HNE staining in these animals.

Overall, our data suggest that St-PGA-CL-BDMC may also protect from ferroptosis thus contributing to nephroprotection in FA-AKI.

### St-PGA-CL-BDMC downregulates NF-κB pathway activation in FA-AKI

**Figure 9:**
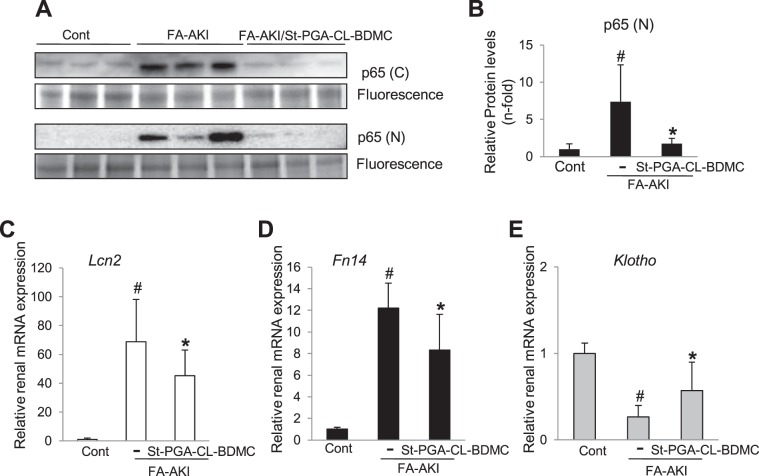
St-PGA-CL-BDMC downregulates NF-κB activation in FA-AKI. (**A**) Representative western blot of p65 assessed in kidney nuclear (p65 (N)) and cytoplasmic (p65 (C)) extracts showing that St-PGA-CL-BDMC prevented the FA-AKI-induced increase in NF-κB levels and nuclear translocation. Protein associated-fluorescence was used as loading control and a representative fluorescent band showed as inverted image. (**B**) Quantification of nuclear p65 (p65(N)) assessed by western blot in the complete set of mice. ^#^p < 0.05 vs control; *p < 0.05 vs FA-AKI. (**C–D**) The Lcn2 and Fn14 relative renal mRNA expression is significantly lower in St-PGA-CL-BDMC-treated FA-AKI mice. ^#^p < 0.001 vs control; *p < 0.05 vs FA-AKI. (**E**) St-PGA-CL-BDMC treatment prevents the FA-AKI-induced reduction in kidney Klotho mRNA levels. ^#^p < 0.001 vs control; *p < 0.05 vs FA-AKI.

Since cell culture studies demonstrated that St-PGA-CL-BDMC prevented NF-κB/p65 nuclear translocation and the subsequent transcription of NF-κB target genes involved in the pathogenesis of AKI (Fig. 4), we explored the impact of St-PGA-CL-BDMC in kidneys from mice with FA-AKI in this regard. Increased renal levels and activation of NF-κB in FA-AKI mice was apparent from higher cytoplasmic as well as nuclear p65 levels, respectively **(**Fig. 9A,B**) (**Supplementary Fig. S5**)**, increased Lcn2 **(**Fig. 9C**)** and Fn14 **(**Fig. 9D**)** mRNA levels, and decreased mRNA expression of the negatively regulated NF-κB target, Klotho **(**Fig. 9E**)**. Remarkably, St-PGA-CL-BDMC prevented these responses (Fig. 9A–E), suggesting that inhibition of NF-κB activation has a role in St-PGA-CL-BDMC-induced nephroprotection.

## Discussion

The lack of effective AKI treatments currently contributes to acute mortality from AKI and chronic complications, such as progression to CKD, and its own associated high mortality risk^35–37^ 24988558^38^. We have synthesized a novel polymer-drug conjugate, St-PGA-CL-BDMC, that provides the beneficial activities of curcuminoids with an improved pharmacokinetic profile afforded by polymer conjugates, and we also characterized its nephroprotective potential in AKI. Furthermore, we identified critical targets of the nephroprotective effect, including NF-κB activation and interference with apoptosis and regulated necrosis pathways.

Curcuminoids are widely used medications in Indian ayurvedic and traditional oriental medicine. Recently, numerous studies have explored their mechanisms of action^11,39^, with curcuminoids discovered to display anti-inflammatory, antiproliferative, and antioxidative properties that may be harnessed to treat acute and chronic human disease. However, curcuminoids exhibit certain disadvantages, including low bioavailability and suboptimal pharmacokinetic properties^15,40^. Nanoscale has been proven to be an appropriate strategy to overcome bioavailability limitations and improve the bioactivity of diet-derived phytochemicals and curcumin^16,17,41,42^. Specifically, the use of polymer therapeutics, and the development of polymer-drug conjugates, in particular, provide longer drug half-lives, improve pharmacokinetic/pharmacodynamic properties, and increase drug accumulation within inflamed/diseased/damaged tissues thanks to the enhanced retention and permeability effect^18,43^. The use of biodegradable polyglutamates as a carrier for AKI therapeutics takes advantage of their natural and significant kidney tropism^23,24^. Macromolecular complexes of polyglutamates, including St-PGA-based nanocarriers and, by extension, the therapeutic polymer assayed in the present work, typically enter cells by an energy-dependent endocytic process^23,24,44^. St-PGA-CL-BDMC is a pH-responsive functional polymer conjugate that allows drug delivery in acidic environments, such as inflamed tissues, e.g. areas of damaged kidney in AKI, or may take the endocytic-lysosomal pathway in native tubular cells also leading to a pH-aided polymer cleavage and drug release^44,45^. The large conjugate size (30–100 nm) does not support glomerular filtration as mechanism of urinary excretion of the unprocessed molecule. Moreover, published detailed pharmacokinetic studies showed plasma clearance of 1.193 ml/h and *in vivo* distribution of St-PGA in healthy mice characterized by efficient renal uptake and a wide organ distribution, including accumulation in immune cells of lymphoid organs^28,29^, which could be potentially important to control deregulated proinflammatory activity during AKI. Kidney St-PGA accumulation was already detected at 30 min, peaked at 8 h and remained significantly retained after 72h^29^, which could account for the *in vivo* St-PGA-CL-BDMC inhibitory effects during AKI. The long half-life of the retained fraction of St-PGA-CL-BD in kidney is consistent with catabolism within tubular cells. St-PGA-CL-BDMC displayed an improved pharmacokinetic profile when compared to unconjugated curcuminoids, as evidenced by the *in vitro* and *in vivo* results obtained herein. *In vitro*, in cultured renal tubular cells, St-PGA-CL-BDMC afforded significant protection from cytotoxicity at concentrations 100-fold lower than the unconjugated “free” form of BDMC and *in vivo*, St-PGA-CL-BDMC prevented AKI at doses 25–50 times lower than those commonly employed in experimental AKI treatment with curcuminoids, which are between 100–200 mg/kg/day when administered orally or through the peritoneal parenteral route^46,47^. These results agree with our previous findings showing the suitability of St-PGA for lysosomotropic drug delivery in tumour cells^23,24^ and now add renal epithelial cells as St-PGA-LC-BDMC targets. As renal tubular cell death is the pivotal pathological event involved in the initiation and propagation of renal damage during AKI^3^, we reasoned that targeting tubular cell death and proinflammatory activity by St-PGA-CL-BDMC may contribute to prevent AKI. Moreover, as cell uptake studies strongly suggested that lysosomotropism may be a general mechanism of St-PGA-CL-BDMC cell uptake, it may be speculated that conjugate uptake by other cell types involved in AKI (e.g endothelium cells or leukocytes) may also contribute to nephroprotection.

Moreover, protection from AKI validates the retro-orbital route for a timely St-PGA-CL-BDMC delivery, thus offering simplicity in the conjugate administration and an easy access of the same to the blood stream. This data suggests a rapid absorption of the conjugate into the bloodstream as a consequence of its high solubility in aqueous medium, which mainly differs from the insoluble nature of free curcuminoids in polar aqueous solvents that preclude the retro-orbital administration of free BDMC for comparative purposes.

Our studies confirmed previously published mechanisms of action of curcuminoids in tissue protection, such as the prevention of NF-κB activation and high antioxidant activities, but also provided novel findings regarding the interference of molecular pathways involved in regulated necrosis. In cultured renal tubular cells, St-PGA-CL-BDMC protected from apoptosis, a primary driver of AKI initiation and amplification^46,48^. This result agrees with the reported antiapoptotic activity of curcuminoids in renal cells, and also demonstrated that St-PGA-CL-BDMC, but not the free forms of BDMC or curcumin, inhibited apoptosis at low equimolar doses, suggesting the enhanced bioavailability when formulated as a polymer-drug conjugate. In this regard, unlike free curcuminoids that enter the cells by passive diffusion, polymer-conjugates require an energy-dependent mechanism for cellular uptake^18^. After endocytosis, the conjugated drug then accumulates in the lysosome thereby avoiding detoxifying mechanisms such as ATP-binding cassette and efflux pumps. Following polymer and/or linker degradation, the drug is then released to the cytosol^45^. Of note, lysosomal cathepsins are involved in lysosome-mediated apoptosis in several pathological processes including renal diseases and AKI, and, as recently reported, in ferroptosis^49,50^. Moreover, previous studies have revealed that curcumin restrains the leakage of hydrolases by an effect that depends on the lysosomal membrane stabilization^51^. This findings suggest that in our system, following to both lysosomotropic delivery of St-PGA-CL-BDMC and its own hydrolityc cleavage, free BDMC also contribute to inhibit the apoptosis promoted by lysosomal enzymes by restoring the lysosomal membrane functionality. In agreement with the *in vitro* results, St-PGA-CL-BDMC also decreased *in vivo* cell death (lower numbers of renal TUNEL positive cells) and tubular caspase-3 activation in FA-AKI. However, TUNEL may also detect caspase-independent cell death, such as forms of regulated necrosis-like necroptosis and ferroptosis implicated in AKI pathogenesis^6,9,52^. Studies have established that curcumin protects hepatocytes and neurons from necroptosis, although kidney cells have not been studied^53,54^. Our results in cultured renal tubular cells demonstrated that St-PGA-CL-BDMC-mediated inhibition of z-VAD/TTI-induced necroptosis, as assessed by the inhibition of cell death and MLKL phosphorylation, add a new perspective in the explanation of the lower severity of cell death observed in AKI mice treated with the St-PGA-CL-BDMC. Unfortunately, difficulties for detection of phosphorylated MLKL in mouse kidney^6,9^ precluded verifying a direct effect of St-PGA-CL-BDMC on AKI-induced necroptosis *in vivo*. Also, St-PGA-CL-BDMC decreased the expression of ferroptosis markers in kidneys from FA-AKI, identifying a previously uncharacterized modulation by curcuminoids of this regulated necrosis cell death mechanism. Recent investigations of our laboratory established that ferroptosis is a crucial mechanism initiating AKI, whereas necroptosis mediates a second, amplificatory wave of cell death^6,9^.

Curcuminoids inhibit activation of NF-κB, a critical proinflammatory transcription factor in kidney disease^32^. St-PGA-CL-BDMC treatment downregulated NF-κB activation and downstream events in cultured renal tubular cells, including expression of genes associated with inflammation (CCL2, CCL5/Fn14) and tubular damage (Lcn2). Moreover, St-PGA-CL-BDMC treatment also inhibited these and other NF-κB-dependent responses in FA-AKI mice concomitant to repression of NF-κB/p65 upregulation and nuclear translocation. Whereas lower levels of HO-1, Lcn2 and PCNA in a context of preserved function may reflect the milder renal injury^55^, the inhibition of FA-AKI-induced Fn14 expression and Klotho suppression could also contribute to the downregulation of AKI-associated inflammation, thus preventing the amplification of injury. In this regard, Fn14 targeting decreases the severity of FA-AKI and limits kidney inflammation and necroptosis^9^, while Klotho has anti-inflammatory, tissue-protective, and anti-fibrotic properties^56^.

In conclusion, we present St-PGA-CL-BDMC as a novel polymer-drug conjugate that displays the potent nephroprotective properties common to other curcuminoids, but with a more favorable pharmacokinetic profile. St-PGA-CL-BDMC protected cultured renal tubular cells at lower concentrations that non-conjugated free curcuminoids and protected from AKI through the inhibition of NF-κB activation and interference with molecular pathways driving cell death through apoptosis and the necroptosis and ferroptosis forms of regulated necrosis. These properties make St-PGA-CL-BDMC a candidate for future clinical studies of AKI prevention and therapy.

## Supplementary information

Supplementary Information.
