# Supplementary material for: Effective Nephroprotection Against Acute Kidney Injury with a Star-Shaped Polyglutamate-Curcuminoid Conjugate
Source: Sci Rep. 2020 Feb 6;10:2056. doi: 10.1038/s41598-020-58974-9 (PMC7005021; doi:10.1038/s41598-020-58974-9)
Supplement: Supplementary file 1 — Supplementary Information. [file 41598_2020_58974_MOESM1_ESM.pdf]

SUPPLEMENTARY INFORMATION FOR:

Effective Nephroprotection Against Acute Kidney Injury with a Star-Shaped Polyglutamate-Curcuminoid Conjugate

Gina Córdoba-David[1], Aroa Duro-Castano[2], Regiane Cardoso Castelo-Branco[1], Cristian González-Guerrero[1], Pablo Cannata[3], Ana B Sanz[1,4], María J. Vicent[2], Alberto Ortiz[1,4], Adrián M. Ramos[1,4*]

1 Laboratory of Nephrology, IIS-Fundación Jiménez Díaz, School of Medicine, UAM, Madrid, Spain

2 Polymer Therapeutics Lab, Centro de Investigación Príncipe Felipe, Valencia, Spain

3 Pathology, IIS-Fundación Jiménez Díaz, School of Medicine, UAM, Madrid, Spain

4 Red de Investigación Renal (REDINREN),

*Corresponding Author

SUPPLEMENTARY MATERIALS AND METHODS

1. Carrier synthesis and characterization

All chemicals were reagent grade, obtained from Aldrich, and used without further purification unless otherwise indicated. BDMC was purchased from TCI. All solvents were of analytical grade and were dried and freshly distilled. Deuterated chloroform-*d1*, DMSO-*d6*, and D_2O were purchased from Deutero GmbH. Preparative SEC was performed using Sephadex G-25 superfine or Sephadex LH-20 superfine from GE. Dialysis was performed in a Millipore ultrafiltration device fitted with a 3, or 5 kDa MWCO regenerated cellulose membrane (Vivaspin®).

NMR spectroscopy. ^{1}H spectra were recorded on a Bruker AC 300 at room temperature and a frequency of 300 and analyzed using the MestreNova 6.2 software.

Gel Permeation Chromatography (GPC) in DMF. For SEC measurements in DMF containing 1g/L of lithium bromide (LiBr) as an additive, a GPC max (Malvern Instruments) autosampler was used with a flow rate of 0.7 mL/min at 60°C as an integrated instrument, including two columns (105/103/102Å porosity) from Tosoh. Viscotek TDA™ 302 triple detector was used as an integrated detection system (Refractive index and Light Scattering), and Calibration was achieved with well-defined poly (methyl methacrylate) (PMMA, 60 kDa) standards, purchased from Polymer Standards Service (PSS)/Mainz Germany. 100 µL of a polymer solution of 8 mg/mL was injected each time.

Dynamic Light Scattering (DLS). DLS measurements were performed using a Malvern ZetaSizer NanoZS instrument, equipped with a 532 nm laser at a fixed scattering angle of 173°. Solutions were sonicated for 10 min, allowed stabilize for 24 h, filtered through a 0.45 µm cellulose membrane filter, and measured. Size distribution was measured (radius, nm) for each polymer in triplicate with n> 3 measurements. For size measurements, polymer solutions were prepared under different conditions (ddH_2O, phosphate buffers, or salt containing solutions, at different concentrations and temperatures). Automatic optimization of beam focusing and attenuation was applied for each sample.

Z-Potential Measurements. Z-potential measurements were performed at 20°C using a Malvern Zetasizer NanoZS instrument, equipped with a 532 nm laser using Disposable folded capillary cells, provided by Malvern Instruments Ltd. (Worcestershire, UK). Polymer solutions were prepared in 1 mM KCl in ddH_2O. The solutions were filtered through a 0.45 µm cellulose membrane filter. Z-potential was measured for each sample per triplicate with n> 3 measurements.

Ultraviolet-Visible spectroscopy (UV-VIS). UV-VIS measurements were performed for curcumin loading characterization using JASCO V-630 spectrophotometer at 25°C with 1.0 cm matched quartz cells and with a spectral bandwidth of 0.5 nm.

SUPPLEMENTARY TABLES

Table S1. GPC and ^{1}H NMR determination of molecular weight distribution, degree of polymerization, and polydispersity of the Star-PGA polymer before benzyl groups deprotection (St-PBLG). ^{a}Data obtained by GPC in DMF/LiBr (1%) at 8mg/mL. ^{b}Data obtained by ^{1}H NMR in TFA-d1 of benzyl protected polyglutamate through the integral ratio of aromatic initiator and alpha chiral glutamate proton signals

Compound	DPtheo	Mn^{a} (kDa)	Mn^{b} (kDa)	DP^{a}	DP^{c}	Đ
**St-PBLG polymer**	150	31.4	37.4	143	171	1.26

Table S2. Rh values obtained by dynamic light scattering of the polymer-conjugate and precursors in water expressed in number mean.

SIZE 1 mg/mL in H_2O Number mean	SIZE (r, nm)	DESVEST (nm)	ERROR (nm)
Star PGA	53.45	11.16	3.94
St-PGA-propargyl (13)	37.84	4.19	2.10
St-PGA-EG (2)N3 (7)	42.88	2.72	1.57
St-PGA-Crosslinked (CL)	40.93	3.58	1.60
St-PGA-CL-BDMC	40.26	2.45	1.42

Table S3. Z-potential of the polymer-conjugate and precursors in 1 mM solution of KCl.

Z-potential 1 mg/mL in KCl	ZP (mV)	DESVEST (mV)	ERROR (mV)
Star PGA	-43.93	3.40	1.96
St-PGA-propargyl (13)	-38.47	2.93	1.69
St-PGA-EG (2)N3 (7)	-47.70	2.86	1.65
St-PGA-Crosslinked (CL)	-45.10	2.97	1.72
St-PGA-CL-BDMC	-38.60	2.17	1.25

SUPPLEMENTARY FIGURE LEGENDS

Supplementary Figure S1. Intracellular delivery and location of St-PGA-CL-BDMC in tubular cells. In vivo confocal images of St-PGA-CL-BDMC (red fluorescence) uptake at 6 h post-treatment in murine tubular cells (MCT). Lysosomes were stained with LysoTracker Green (green fluorescence). The merged image shows colocalization of the conjugate and tracked lysosomes as indicated by the white arrows. Original magnification x 480. Scale bar 25 µm.

Supplementary Figure S2. Histological assessment of kidney lesions in AKI or AKI-St-PGA-CL-BDMC mice. Kidney tissue sections from mice with folic acid-induced AKI (AKI), AKI and cotreatment with St-PGA-CL-BDMC (AKI/ St-PGA-CL-BDMC) and vehicle treated (Cont) were stained with H&E. AKI mice showed extended areas of tubular necrosis (asterisks) **(b, c, d)** whereas the degree of the injury was lower in AKI mice cotreated with St-PGA-CL-BDMC **(c,e)**. Typical signs of tubular injury are indicated as follows: tubules with necrosis (asterisks) characterized by cell detachment and intratubular cell debris (red arrows); apoptosis (black arrows); calcifications reminiscents of previous necrosis (white arrows), giant nuclei (yellow arrow) and mitosis (black arrowhead). In figure **d**, the square delimitates an area of intense necrosis displaying detached cells in the tubular lumina. **a, c, e)** Original microphotographs displayed in **Fig.5C. b, d, f)** Extended set of images showing pathological markers in kidney tissue representative of AKI and AKI/St-PGA-CL-BDMC mice.

Supplementary Figure S3. Original western blot images for pc-JUN detection in kidney nuclear protein extracts. Samples were separated in 8% SDS-PAGE. After protein transfer, the membranes were stained with Ponceau Red and probed for p-cJUN detection.To ensure reliable comparison between the different groups of mice, samples of each experimental conditions were run in paralallel and transfered proteins of interest developed at the same time against the same control samples. Vertical dashed lines separate controls from AKI samples.

Supplementary Figure S4. Original western blot images for HO-1 and cleaved IL-33 detection in kidney total protein extracts. A) Representative samples from mice of each experimental condition for results presentation were subjected to 12% SDS-PGE. Immobilized proteins were stained with Ponceau Red and the membrane divided for HO-1 and ERK2 (protein loading control) detection. The lower piece of the membrane was reprobed to detect cleaved IL-33. The panel on the upper right allows verifying changes introduced in the same membrane along the entire procedure. Vertical dashed lines separate controls from AKI samples. **B)** Western blots for quantification of HO-1 and ERK2 (protein loading control) in the entire sets of samples from either mice with AKI (AKI) or mice with AKI cotreated with St-PGA-CL-BDMC (AKI/St-PGA-CL-BDMC). To ensure reliable comparison between the different groups of mice, samples of each experimental conditions were run in paralallel and transfered target proteins developed at the same time against the same control samples.

Supplementary Figure S5. Original western blot images of NF-κB/p65 detection in kidney nuclear or cytoplasmic protein extracts. A-B) Samples were separated by 8-15% SDS-PAGE and the total fluorescence in each line measured as protein loading control. Transfered proteins were also visualized by Ponceau Red staining. Membranes were cut and developed for NF-κB/p65. The panel on the right allows verifying changes introduced in the same membrane along the entire procedure. Vertical dashed lines separate controls from AKI samples. **C)** Quantification of the protein-associated fluorescence in nuclear (left table) and cytoplasmic (right table) samples. **D)** Original western blot images for NF-κB/p65 detection and quantification in nuclear extracts from the entire set of animals with AKI (AKI) or that were cotreated with St-PGA-CL-BDMC (AKI/St-PGA-CL-BDMC). After protein separation by 12% SDS-PAGE, transfered proteins were detected by Ponceau Red staining, then transfered to allow simultaneous immudetection of the target protein (NF-κB/p65, top membranes 1 and 2) and the Histone 3 as protein loading control (bottom membranes 1 and 2) in AKI or AKI/St-PGA-CL-BDMC mice against the same control samples. The panel on the upper right allows verifying changes introduced in the same membrane along the entire procedure. Vertical dashed lines separate controls from AKI samples.

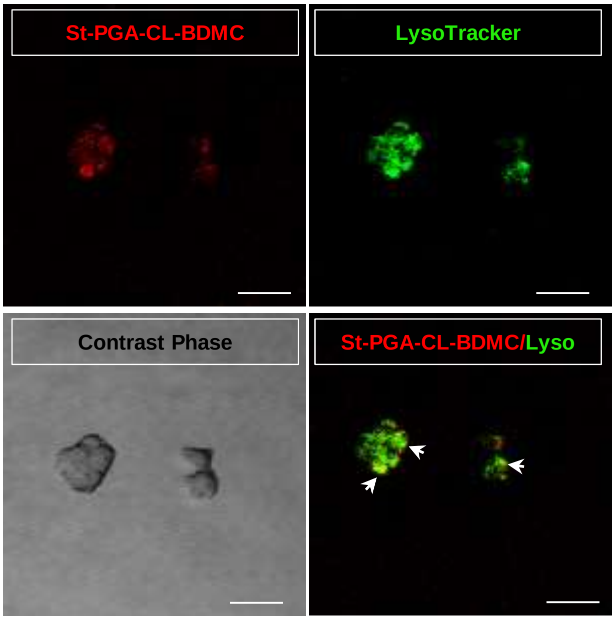

Supplementary. Fig. S1

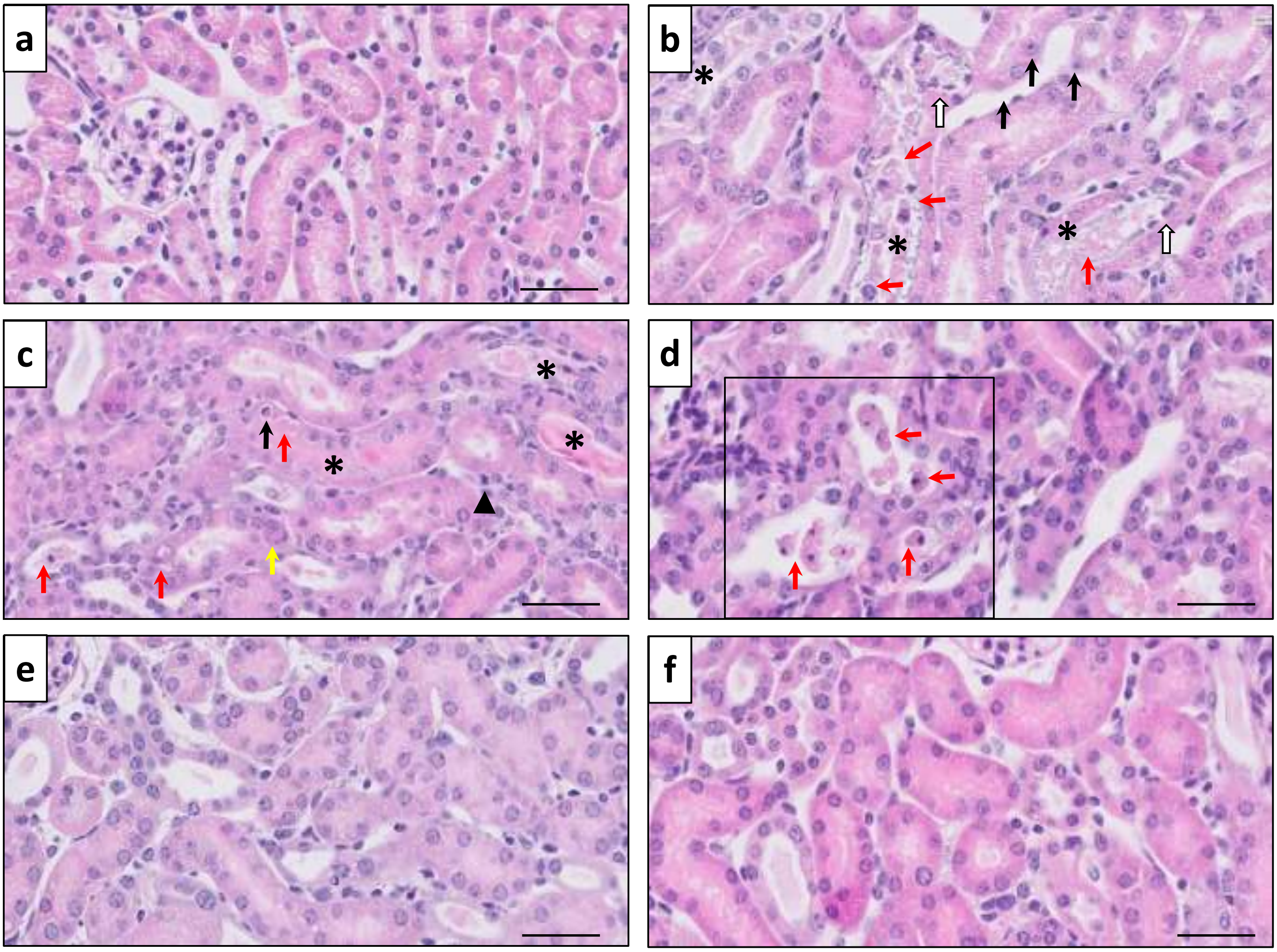

Supplementary. Fig. S2

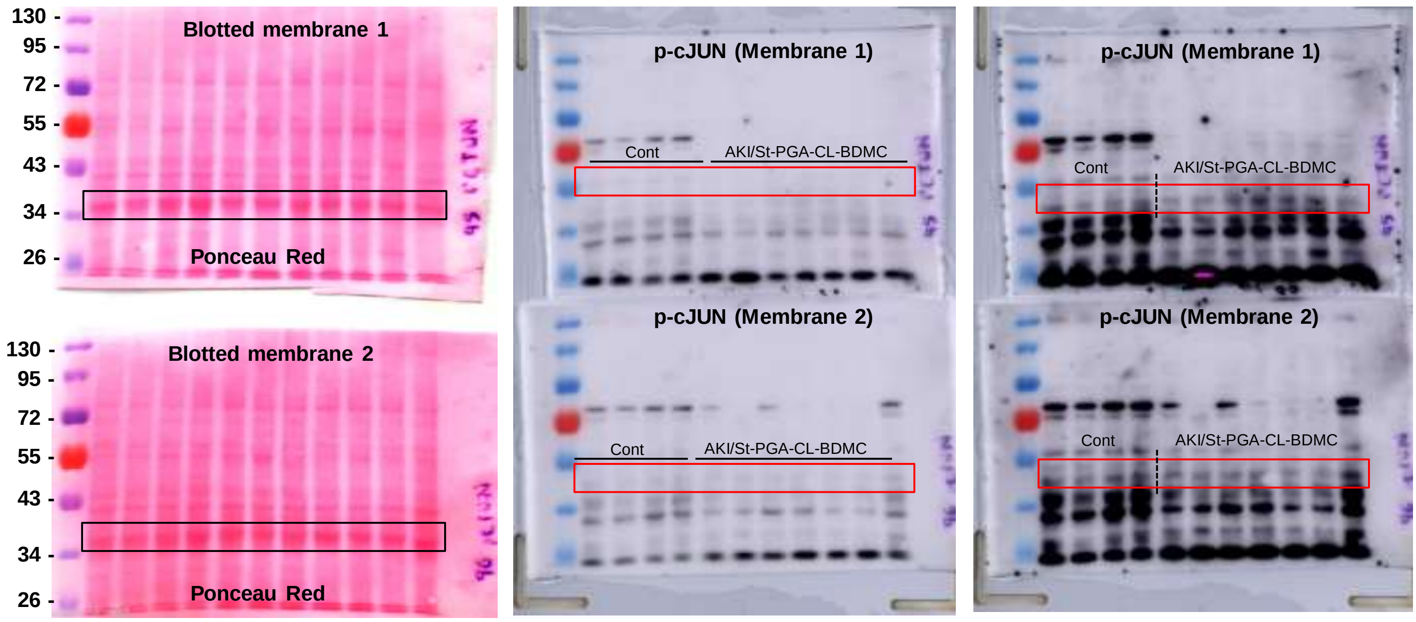

Full membranes and selected areas for the protein loading controls

Normally exposed membranes

Overexposed membranes and selected bands for result presentation in Fig.7C

Supplementary. Fig. S3

A

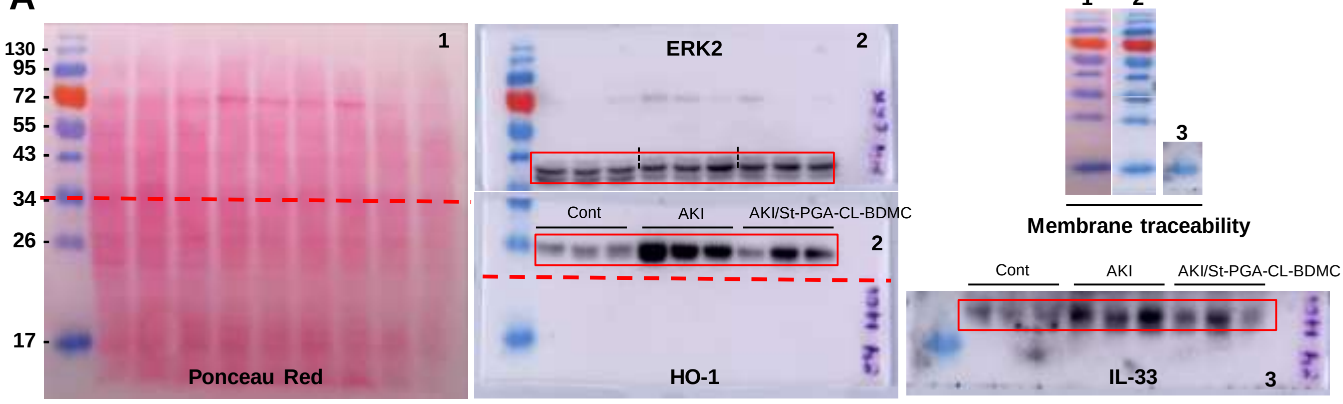

Full membrane (uncut)

Cut membranes and cropped bands
(selected set of samples for results presentation in Fig.8B)

B

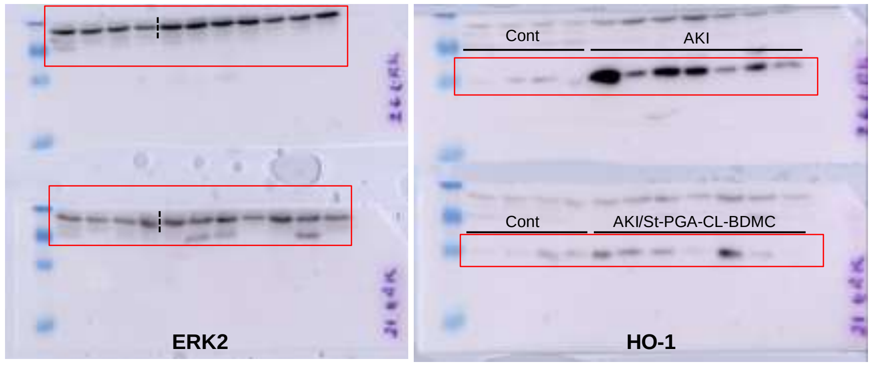

Cut membranes and selected bands (extended set of samples for quantification)

Supplementary. Fig. S4

A

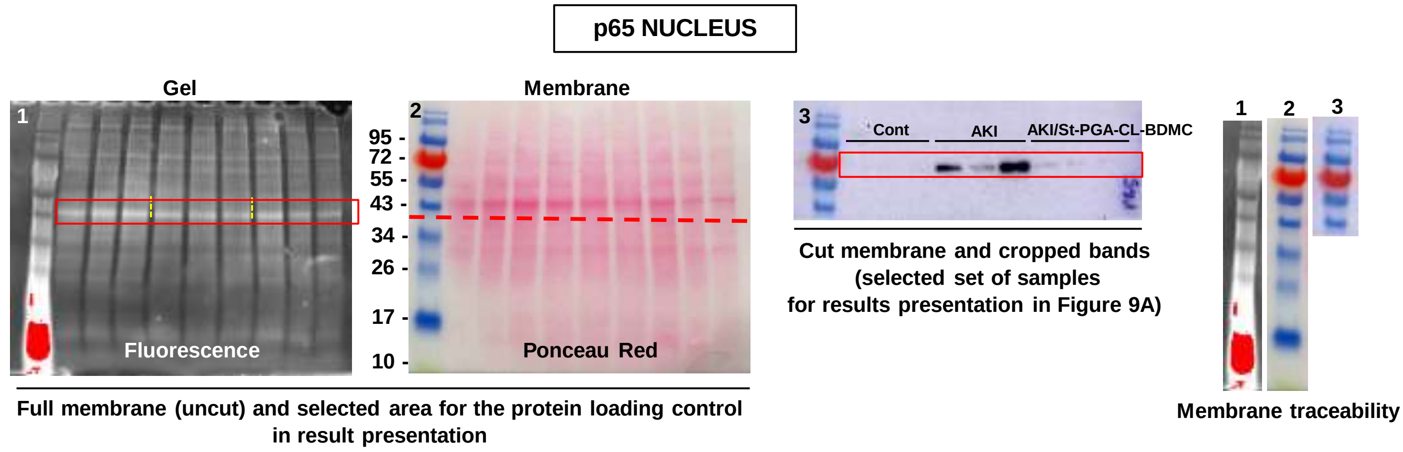

B

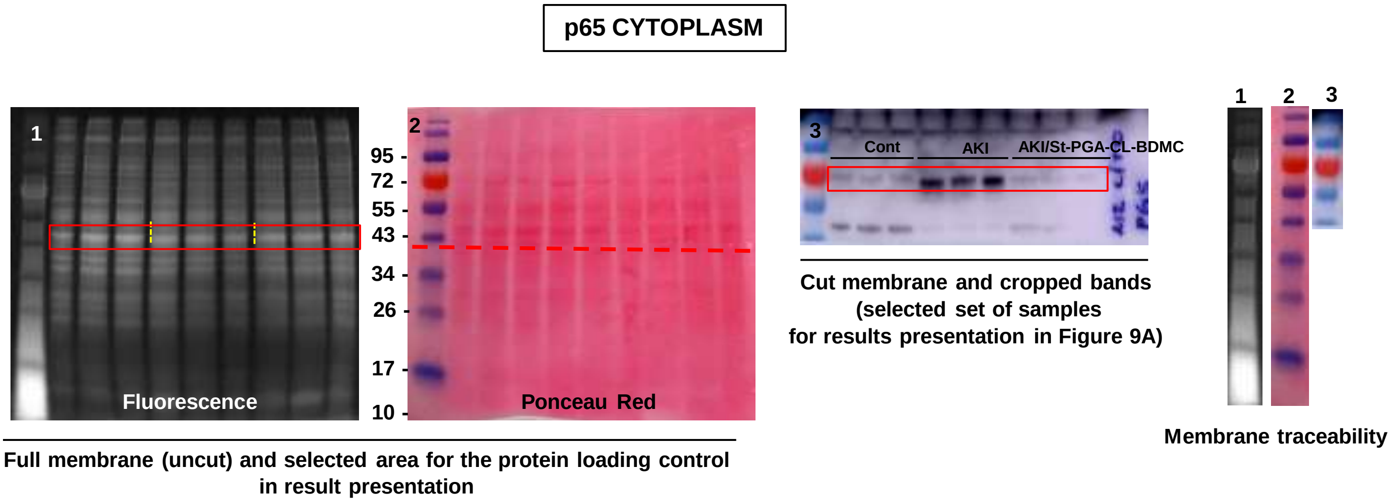

C

NUCLEAR SAMPLES	Fluorescence (full line)	
Treatment	Adj. Vol. (INT/mm2)	Relative value
Cont 1	78.621.875.156.363	1,0
Cont 2	81.015.323.568.433	1,0
Cont 3	81.030.024.480.002	1,0
AKI 1	69.944.837.113.774	0,9
AKI 2	72.451.692.557.946	0,9
AKI 3	73.010.327.197.558	0,9
AKI/St-PGA-CL-BDMC 1	89.671.360.308.781	1,1
AKI/St-PGA-CL-BDMC 2	74.839.540.622.752	1,0
AKI/St-PGA-CL-BDMC 3	60.520.152.711.405	0,8

CYTOPLASMIC SAMPLES	Fluorescence (full line)	
Treatment	Adj. Vol. (INT/mm2)	Relative value
Cont 1	93.372.489.807.060	1,0
Cont 2	111.852.235.692.356	1,2
Cont 3	108.621.535.364.277	1,2
AKI 1	107.504.966.128.462	1,2
AKI 2	87.186.906.253.666	0,9
AKI 3	76.434.239.506.255	0,8
AKI/St-PGA-CL-BDMC 1	85.570.506.024.514	0,9
AKI/St-PGA-CL-BDMC 2	96.157.962.527.629	1,0
AKI/St-PGA-CL-BDMC 3	106.966.632.747.683	1,1

Supplementary. Fig. S5

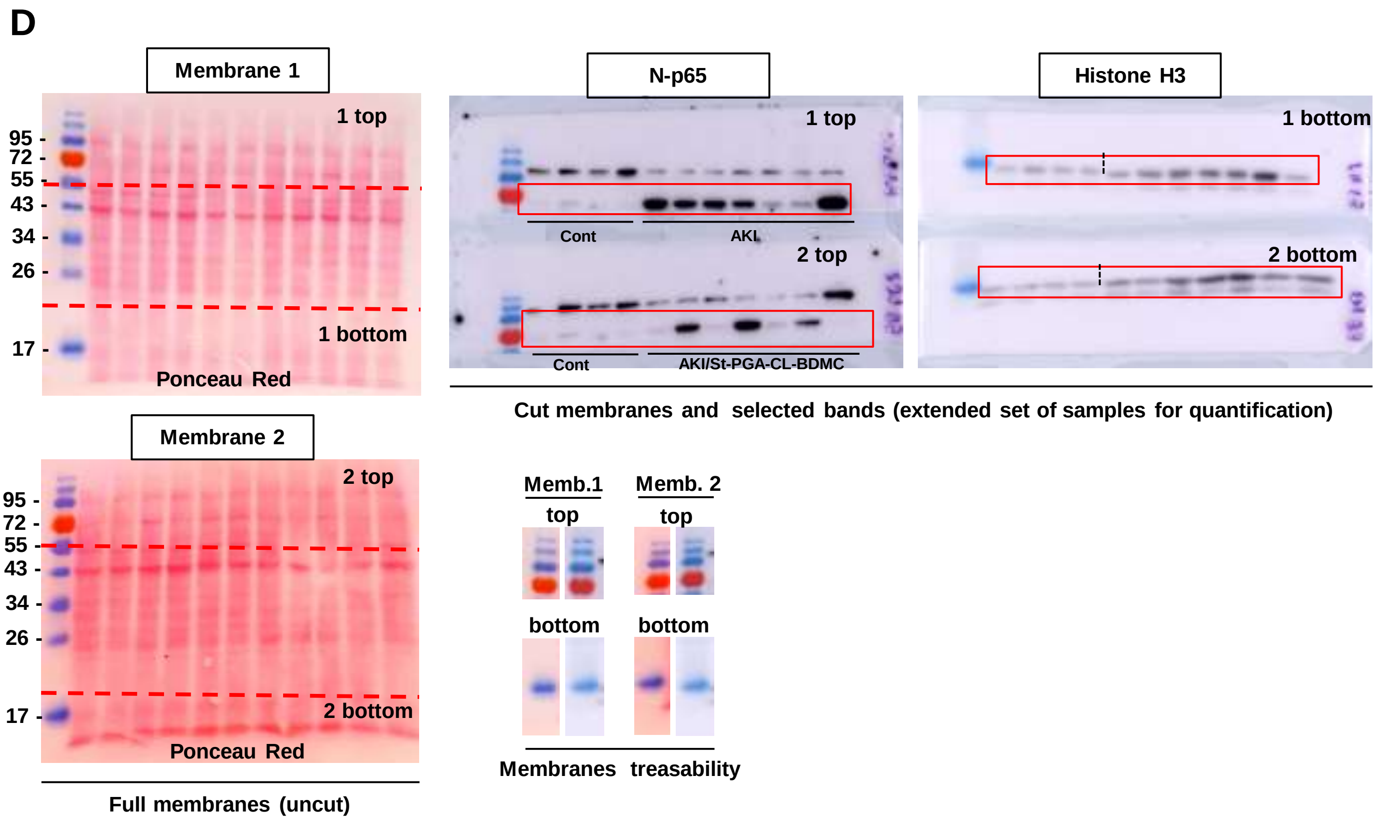

Supplementary. Fig. S5 (cont)
